# Abnormal brain functional network dynamics in sleep‐related hypermotor epilepsy

**DOI:** 10.1111/cns.14048

**Published:** 2022-12-12

**Authors:** Xinyue Wan, Pengfei Zhang, Weina Wang, Xintong Wu, Qiaoyue Tan, Xiaorui Su, Simin Zhang, Xibiao Yang, Shuang Li, Hanbing Shao, Qiang Yue, Qiyong Gong

**Affiliations:** ^1^ Huaxi MR Research Center (HMRRC), Department of Radiology West China Hospital of Sichuan University Chengdu China; ^2^ Department of Radiology, Huashan Hospital Fudan University Shanghai China; ^3^ Second Clinical School Lanzhou University Lanzhou China; ^4^ Department of Magnetic Resonance Lanzhou University Second Hospital Lanzhou China; ^5^ Department of Radiology, The First Affiliated Hospital, College of Medicine Zhejiang University Hangzhou China; ^6^ Department of Neurology West China Hospital of Sichuan University Chengdu China; ^7^ Department of Radiology West China Hospital of Sichuan University Chengdu China; ^8^ Research Unit of Psychoradiology Chinese Academy of Medical Sciences Chengdu China; ^9^ Functional and Molecular Imaging Key Laboratory of Sichuan Province Chengdu China; ^10^ Department of Radiology West China Xiamen Hospital of Sichuan University Xiamen Fujian China

**Keywords:** dynamic functional network connectivity, independent component analysis, network‐based statistics, resting‐state functional magnetic resonance imaging, sleep‐related hypermotor epilepsy

## Abstract

**Aims:**

This study aimed to use resting‐state functional magnetic resonance imaging (rs‐fMRI) to determine the temporal features of functional connectivity states and changes in connectivity strength in sleep‐related hypermotor epilepsy (SHE).

**Methods:**

High‐resolution T1 and rs‐fMRI scanning were performed on all the subjects. We used a sliding‐window approach to construct a dynamic functional connectivity (dFC) network. The k‐means clustering method was performed to analyze specific FC states and related temporal properties. Finally, the connectivity strength between the components was analyzed using network‐based statistics (NBS) analysis. The correlations between the abovementioned measures and disease duration were analyzed.

**Results:**

After k‐means clustering, the SHE patients mainly exhibited two dFC states. The frequency of state 1 was higher, which was characterized by stronger connections within the networks; state 2 occurred at a relatively low frequency, characterized by stronger connections between networks. SHE patients had greater fractional time and a mean dwell time in state 2 and had a larger number of state transitions. The NBS results showed that SHE patients had increased connectivity strength between networks. None of the properties was correlated with illness duration among patients with SHE.

**Conclusion:**

The patterns of dFC patterns may represent an adaptive and protective mode of the brain to deal with epileptic seizures.

## Graphical abstract

After k‐means clustering, the SHE patients mainly have two dFC states. The frequency of state 1 was higher, which is characterized by stronger connections within the network, including executive control, default mode, sensorimotor, and visual network; state 2 occurred at a relatively low frequency, characterized by stronger connections between networks, including sensorimotor, visual, and auditory networks. It turns out that SHE patients showed preference in state 2.

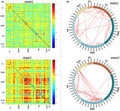

## 1 INTRODUCTION

Sleep‐related hypermotor epilepsy (SHE), which was formerly known as nocturnal frontal lobe epilepsy (NFLE), is characterized by brief, clustering, and hyperkinetic seizures that mainly occur during non‐rapid eye movement sleep.^1^ The prevalence of SHE is 1.8 per 100,000, and most patients have no family history or a clear etiology.^2^ Previous studies have shown that epilepsy is a brain network disorder.^3^, ^4^, ^5^ The extensive application of neuroimaging in epilepsy research in recent years provides a foundation for understanding epilepsy networks. The current view is that focal epileptic seizures are caused by complex coordination between large‐scale epilepsy networks.^6^ As a rare type of focal epilepsy, some previous studies have also found that the brains of SHE patients are widely affected rather than confined to a certain region.^7^, ^8^ Therefore, studying the epilepsy network of SHE will help us further understand its mechanisms.

The findings of conventional magnetic resonance imaging (MRI) of SHE patients were previously considered to be negative in that they did not reveal structural abnormalities. However, with the development of neuroimaging, a growing number of methods have helped us find differences in brain function that cannot be observed by conventional MRI. Resting‐state functional magnetic resonance imaging (rs‐fMRI) is the most common method to explore functional connectivity by calculating the Pearson correlation coefficient between time series, which characterizes the synergistic activity in the brain. It has been widely used to explore the influence and mechanism of the epileptic network.^9^, ^10^, ^11^ There are many studies of epilepsy that have found abnormal functional connectivity (FC) in multiple brain regions or networks, such as the basal ganglia, cerebral cortex, and default mode network.^7^, ^12^, ^13^, ^14^ In general, all these abovementioned studies assumed that functional connectivity is stationary during the scanning process, but in fact, the brain connectivity is highly dynamic in the resting state.^15^

Static functional connection analysis (sFC) ignores the temporal characteristics of functional connectivity.^16^, ^17^, ^18^ Given this, the concept of Dynamic Functional Connectivity (dFC) was brought forward, which takes into account the dynamic alterations of brain functional activities over time,^19^ and it has been applied to explore many types of epilepsy^20^, ^21^ and other diseases.^22^, ^23^ At present, it is still not known what the changes are of the dFC and temporal features of the dFC state in SHE patients. Applying dFC analysis to patients with SHE, a rare epilepsy with seizures, helps provide finer and more informative information on neural alteration processes and reveals the functional coordination and adaptation of brain networks, helping us further discover time‐varying evidence of abnormal brain function in patients with SHE.

Based on the rs‐fMRI data, a group independent component analysis (GICA) was performed to obtain independent components (ICs). We used the sliding window method to construct the dFC matrix of the SHE patients and the healthy controls (HCs). Then, using cluster analysis, we explored the differences in the specific connectivity states and related temporal features of the SHE patients. According to the different states, we used network‐based statistics (NBS) to probe into the abnormal links. Finally, correlations between the indicators and clinical characteristics were performed. We hypothesized there would be abnormal temporal properties in dFC states and dynamic network properties, which would be correlated with clinical characteristics.

## 2 METHODS

### 2.1 Participants and statistical analyses

SHE patients from the Epilepsy Center of West China Hospital of Sichuan University were assessed by two neurologists (X. Wu and W. Liu) between December 2013 and March 2021. According to the International League Against Epilepsy Classification and Terminology (ILAE 2017), the diagnosis was based on clinical history, seizure semiology, and audio–video recording. The control group was recruited through posted advertisements. The inclusion criteria for all participants were (i) being 18 to 60 years old; (ii) negative brain MRI findings; (iii) no history of other neuropsychiatric or organic diseases; (iv) no MR contraindications; and (v) being right‐handed. The patients must have had at least one hypermotor event during sleep. This study was approved by the local ethical committee of the West China Hospital of Sichuan University, and informed consent was obtained from all participants.

All the statistical analyses were performed using the open‐source R package v3.6. The Shapiro–Wilk test was performed to test the normality of all the continuous variables. For non‐normal data comparisons, the non‐parametric permutation tests were used. Group differences were assessed using the two‐sample t‐test for age and the chi‐square test for gender.

### 2.2 MRI acquisition

High‐resolution T1‐weighted MRI and rs‐fMRI data were acquired with a Siemens 3 T scanner (Trio Tim, Erlangen, Germany), using an 8‐channel phased array head coil. All participants were asked to relax and stay awake with their eyes closed. During the scan, earplugs were used to reduce noise and pads were used to reduce head motion. The scan parameters for the T1 images were as follows: Magnetization Prepared Rapid Gradient Echo (MPRAGE) sequence; repetition time/echo time (TR/TE) = 2250/2.6 ms; flip angle = 9°; slice thickness = 1 mm; matrix = 256 × 256; and field of view 256 × 256 mm^2^. The rs‐fMRI acquisitions were performed using a gradient echo‐planar imaging (EPI) sequence with the following parameters: TR/TE = 2000/35 ms; flip angle = 68°; voxel size = 3.3 × 3.3 × 3.0 mm^3^; slice thickness = 3 mm; matrix size = 64 × 64; field of view = 208 × 208 mm^2^; time points = 220; and acquisition time = 7.20 min. An experienced radiologist checked the participants' MR images to ensure that each patient's MRI was negative.

### 2.3 Data preprocessing

The resting state fMRI data were preprocessed using the toolbox for Data Processing & Analysis of Brain Imaging (DPABI, http://rfmri.org/dpabi).^24^ The preprocessing included removing the first 10 volumes of rs‐fMRI data to reduce equilibration effects; slice‐time correction; realigning to reduce displacement between volumes; co‐registering to their structural images; segmenting of the transformed structural images; normalization into the standard Montreal Neurological Institute (MNI) space; resampling the functional images to 3 × 3 × 3 mm^3^ resolution; and smoothing with a 6‐mm full width half max (FWHM) Gaussian kernel. Participants with a mean framewise displacement (FD) larger than 0.2 mm, a maximum head displacement >1.5 mm, or a maximum rotation more than 1.5° were excluded.

### 2.4 GICA analysis and identification of intrinsic connectivity networks

Figure S1 shows a flowchart of the analysis process. We performed spatial group independent component analysis (GICA) with the GIFT toolbox (http://milab.mrn.org/software/gift/) to create intrinsic connectivity networks. First, we used principal components analysis (PCA) of the subject‐specific data to reduce them to 120 principal components (PCs).^17^ Then, we used the expectation maximization (EM) algorithm to further reduce the group data to 100 PCs. To ensure stability, the Infomax algorithm was repeated 20 times in ICASSO^25^ (max cluster size = 100, min cluster size = 80), using the one‐sample *t*‐test for each spatial map to acquire a *t*‐map with a threshold of *t* > mean (*μ*) + 4SD (*σ*) (*μ* represents the mean value of all the voxels in each *t*‐map; *σ* represents the SD of the values of each IC).^26^

Based on previous studies,^17^ 51 of the 100 independent components were identified. These ICs met the following criteria^17^: (i) exhibiting peak activity in gray matter; (ii) having low spatial overlap among vascular, ventricular, motion, and susceptibility artifacts; and (iii) having time courses dominated by low‐frequency fluctuations.

After identifying the ICs, the post‐processing was conducted, which mainly involved linear detrending, despiking, a fifth‐order Butterworth low‐pass filtering with a high frequency cut‐off of 0.15 Hz, and multiple regression of the six realignment parameters.

### 2.5 dFC estimation and states analysis

The dFC of each subject was calculated using a sliding‐window approach.^17^, ^19^ We used a Gaussian (*σ* = 3 TRs) function to create a tapered window with a width of 22 TRs (44 s), which can provide a good balance between the quality of the connectivity estimation and the ability to explore the dynamics.^17^, ^27^, ^28^ The window was slided step‐wise by 1 TR over the scan time, resulting in 188 windows. For each window, a regularized precision matrix was used to calculate 51 × 51 paired covariance matrices. We applied the L1 norm penalty in the LASSO framework with 100 repetitions to improve sparsity in the estimations.^29^ Then, the FC matrices were converted to *z*‐values using a Fisher's *z*‐transformation, regressed out for age, gender, and the mean FD.

Next, an FC matrix for all the windows of all the subjects was used to identify the dFC state. In this process, a *k*‐means clustering method was applied, and it was repeated 100 times to ensure the minimum bias of initial random selection of cluster centroids. The similarity between each FC matrix and the cluster centroids was estimated using the Manhattan distance (L1 distance). There are many ways to calculate the *k*‐value (from 2 to 10). In addition, we performed the cluster number validity analysis. According to the silhouette statistic,^30^ the optimal *k*‐value in this study was eventually determined to be 2 (Figure S2), where each cluster centroids of all the subjects represented a recurring dFC pattern. Then, the obtained group centroid was used as the initial centroid to cluster the windowed dFC for each subject.

We used the Brain Connectivity Toolbox(www.brainconnectivity‐toolbox.net) with a normal Louvain community detection algorithm to calculate the modularity index Q of 51 ICs, which can describe the integration level of each state.^31^ Then, three state transition metrics were applied to assess the temporal properties of the FC states: (a) fractional windows, i.e., the mean proportion of time spent in each state; (b) the mean dwell time, which represents the average number of consecutive windows in a state before changing to the other state; and (c) the number of transitions, measured as the number of times that states changed from one to the other. To calculate differences in temporal properties between the patients' group and healthy control group, nonparametric permutation tests (10,000 iterations) were applied^23^ (*p* < 0.05, using a false discovery rate (FDR) correction).

We repeated the abovementioned steps using a tapered window with a width of 30 TRs (60s) to evaluate the consistency and validity of the *k*‐means clustering at different window sizes. After that, Pearson's correlation analysis was performed between the cluster centroids under different window sizes. The correlation coefficients indicated the similarity between the results to find the states with higher correlation to the primary analysis.

### 2.6 Network‐based statistical analysis of each state

For the two states, a network‐based statistical analysis (NBS) was used to find the component pairs in which FC changed in the SHE patients.^32^ This is a nonparametric statistical method that identifies closely connected subnetworks within a larger network. First, to test the significant difference of edges between the two groups, a two‐sample *t*‐test was performed on the functional connectivity (i.e., edges) between the components in state 1 and state 2. In this step, the corresponding *t*‐statistic and *p*‐values of the edges were obtained. Then, the threshold of the edge level was set at *p* = 0.05 to obtain the initial subnetwork. The empirical zero distribution of the initial subnetwork was acquired after permutation test (10,000 times) to evaluate the statistical significance of the observed subnetwork (*p* < 0.05 was the criterion). Age and gender were included as covariates in the abovementioned process.

### 2.7 Correlational analyses

Considering that the dynamic parameters of this study were not normal, Spearman's correlations were preformed between illness duration and altered temporal properties, with age and gender as covariates. The statistical significance threshold was set at *p* ≤ 0.05.

## 3 RESULTS

### 3.1 Demographic and clinical characteristics

Fifty‐four SHE patients and 50 HCs were concluded in this study. Table 1 summarizes the clinical and demographic data. No significant difference was found in age (*p* = 0.71) and gender (*p* = 0.26).

**TABLE 1:** Clinical and demographic characteristics in patient with SHE and healthy control subjects.

	SHE	HC	*p*
Number	54	50	–
Age (years)	29.56 ± 9.22	28.88 ± 8.91	0.71
Gender (male/female)	34/20	26/24	0.26
Duration (years)	8.49 ± 8.68	–	–
Antiepileptic therapy (+/−)	42/12	–	–
Single drug/multiple drugs	24/18	–	–

Abbreviations: HC, healthy control; SHE, sleep‐related hypermotor epilepsy.

### 3.2 Intrinsic connectivity networks

**FIGURE 1:**
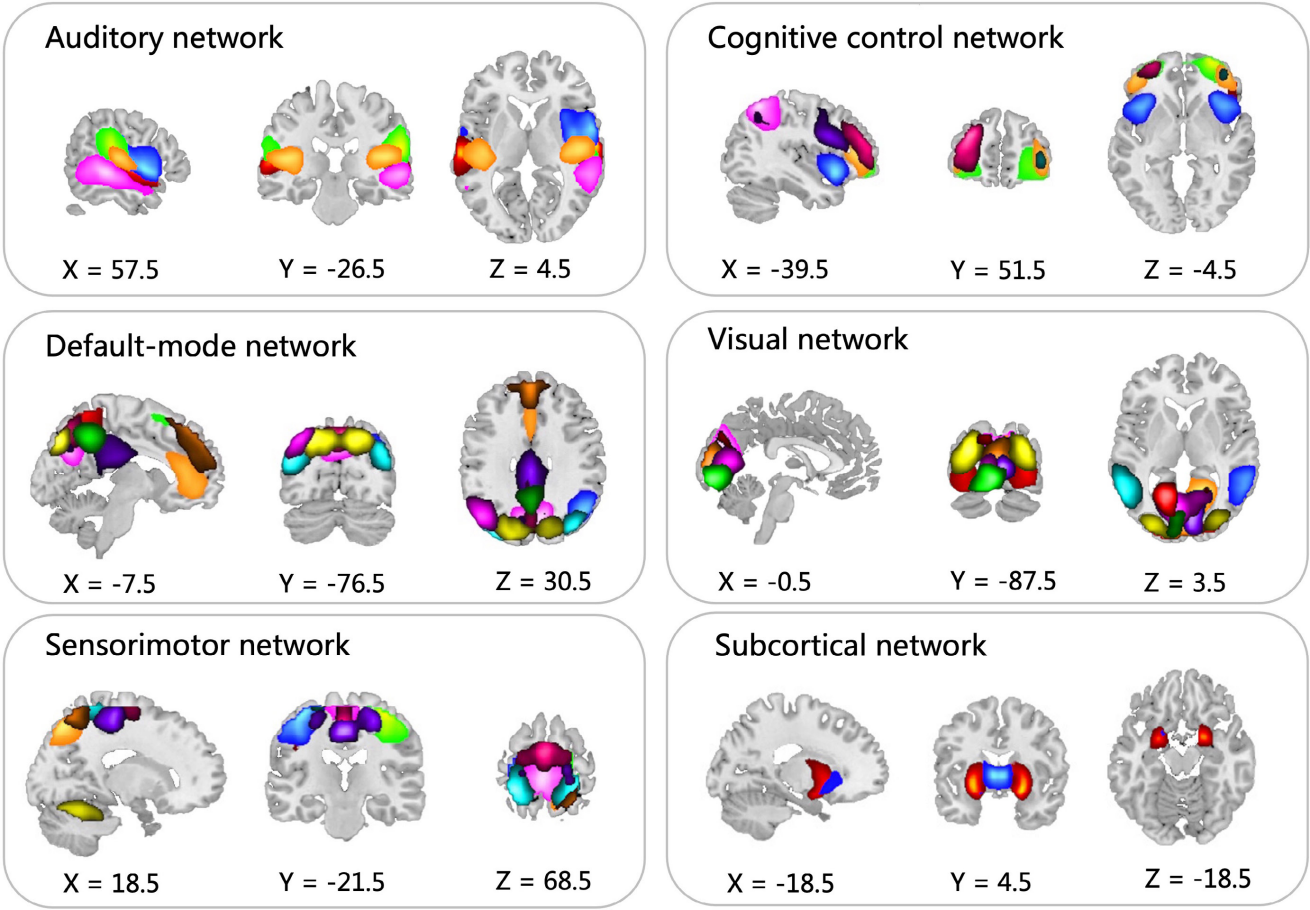
The spatial maps of 51 independent components. *Note*: Fifty‐one components were divided into six instinct networks. They were auditory (AUD), sensorimotor (SMN), subcortical (SC), visual (VN), cognitive control (CC), and default mode (DMN) network. The positions of 51 different components are indicated with different colors.

We sorted 51 ICs into six functional networks: auditory (AUD), sensorimotor (SMN), subcortical (SC), visual (VN), cognitive control (CC), and default mode (DMN) network (Figure 1, Figure S3). The peak coordinates of ICs are shown in Table S1.

### 3.3 Clustering analysis and functional connectivity strength of dFC states

**FIGURE 2:**
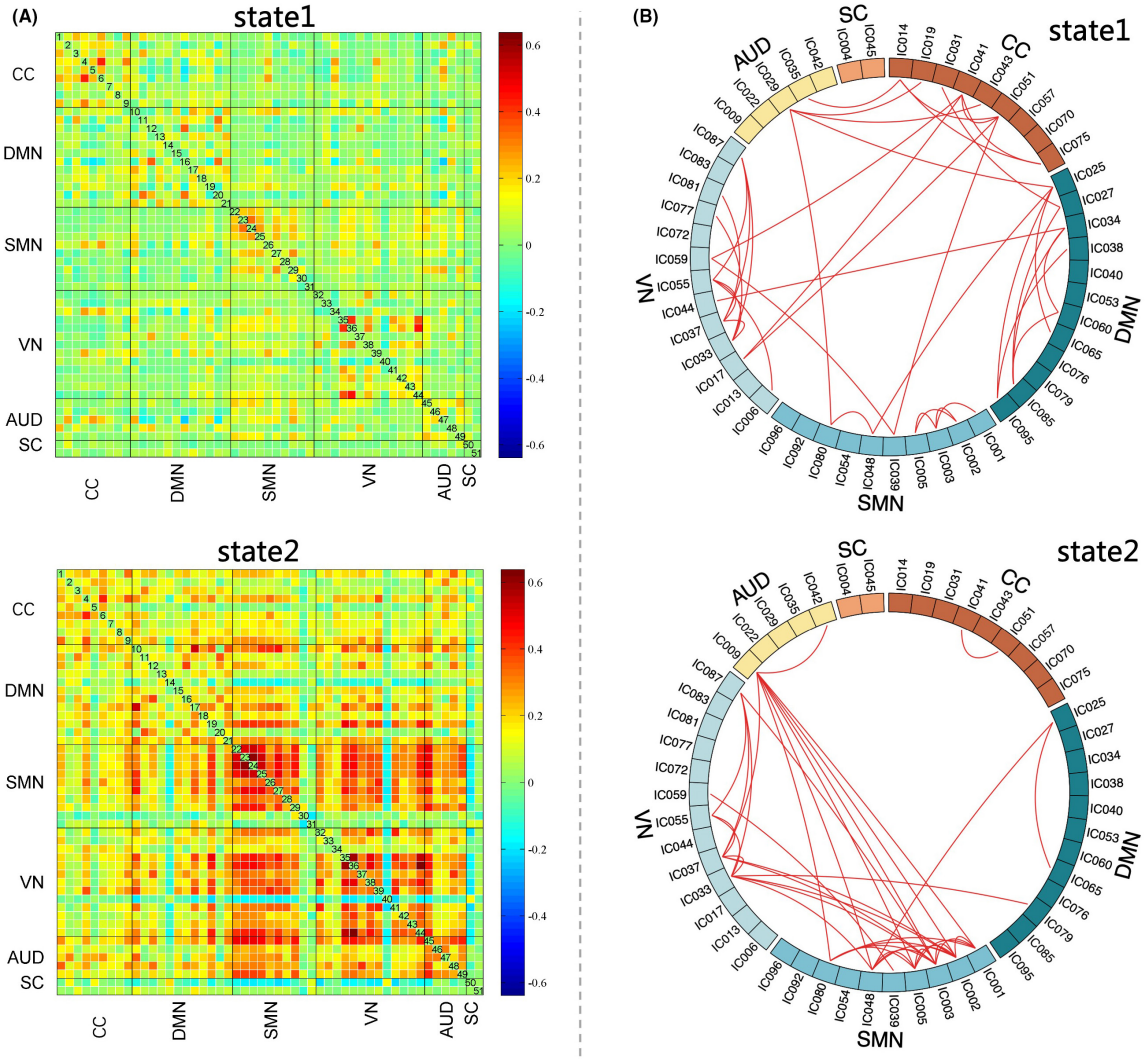
Two dynamic functional connectivity pattern of all subjects. *Note*: A. Cluster centroids for each state. B. The top 3% of the strongest FC in each state. The red lines mean positive connections. AUD, auditory network; CC, cognitive control network; DMN, default mode network; SMN, sensorimotor network; SC, subcortical network; VN, visual network.

**FIGURE 3:**
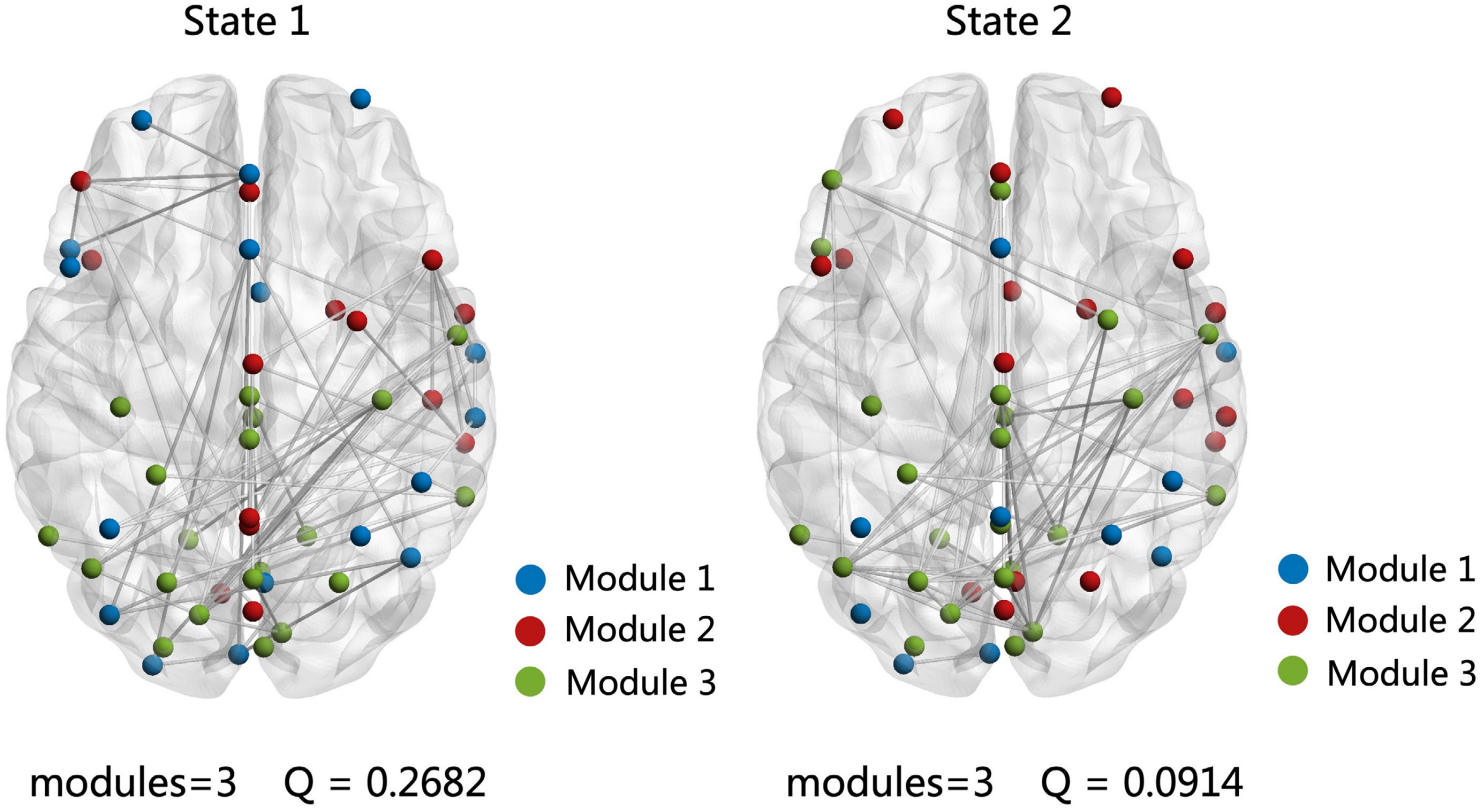
Results of modular analysis for each state. *Note*: State 1 (*Q* = 0.2682) had three modules: module 1 = DMN and CCN, module 2 = CCN, DMN, and VN, module 3 = SMN, VN, and AUD; State 2 (*Q* = 0.0914) was also divided into three modules: module 1 = DMN, CCN, and VN, module 2 = CCN and DMN, module 3 = SMN, VN, and AUD. The index *Q* was used to measure the degree of modularity to evaluate the functional integration within modules and separation between modules. The larger *Q* values indicate a greater tendency to organized ICs into different modules. AUD, auditory network; CC, cognitive control network; DMN, default mode network SMN, sensorimotor network; SC, subcortical network; VN, visual network.

Using the *k*‐means clustering method, two reoccurring state patterns were identified in our study. Figure 2A showed two cluster centroids of all participants which represent two reoccurring FC states. The top 3% of FC matrix in each state is shown in Figure 2B. As the figure shows, the frequency of state 1 was relatively high at the perception of 73. The top 3% of the strongest FC were mainly positive connections within network, including CCN, DMN, VN, and SMN. A small number of positive connections were between networks. Meanwhile, state 2 occupied 27% of the windows. The top 3% of FC were positive connections between AUD, SMN, and VN. There were some positive connections within sensory related network (SMN, VN). Compared with state 1, which was characterized by widely sparse weak connectivity, state 2 showed the relatively stronger connectivity. After modular analysis, this study found different patterns of separation and integration in two states (Figure 3). State 1 had a higher *Q* value, with three functional modules, and represented a stronger separation. Module 1 involved DMN and CCN; module 2 involved CCN, DMN, and VN; and the last module involved SMN, VN, and AUD. State 2 was also divided into three modules, which represented obvious integration. The first module mainly referred to DMN, CCN, and VN; the second module involved CCN and DMN; and the third module contained SMN, VN, and AUD.

### 3.4 Group differences in temporal properties

**FIGURE 4:**
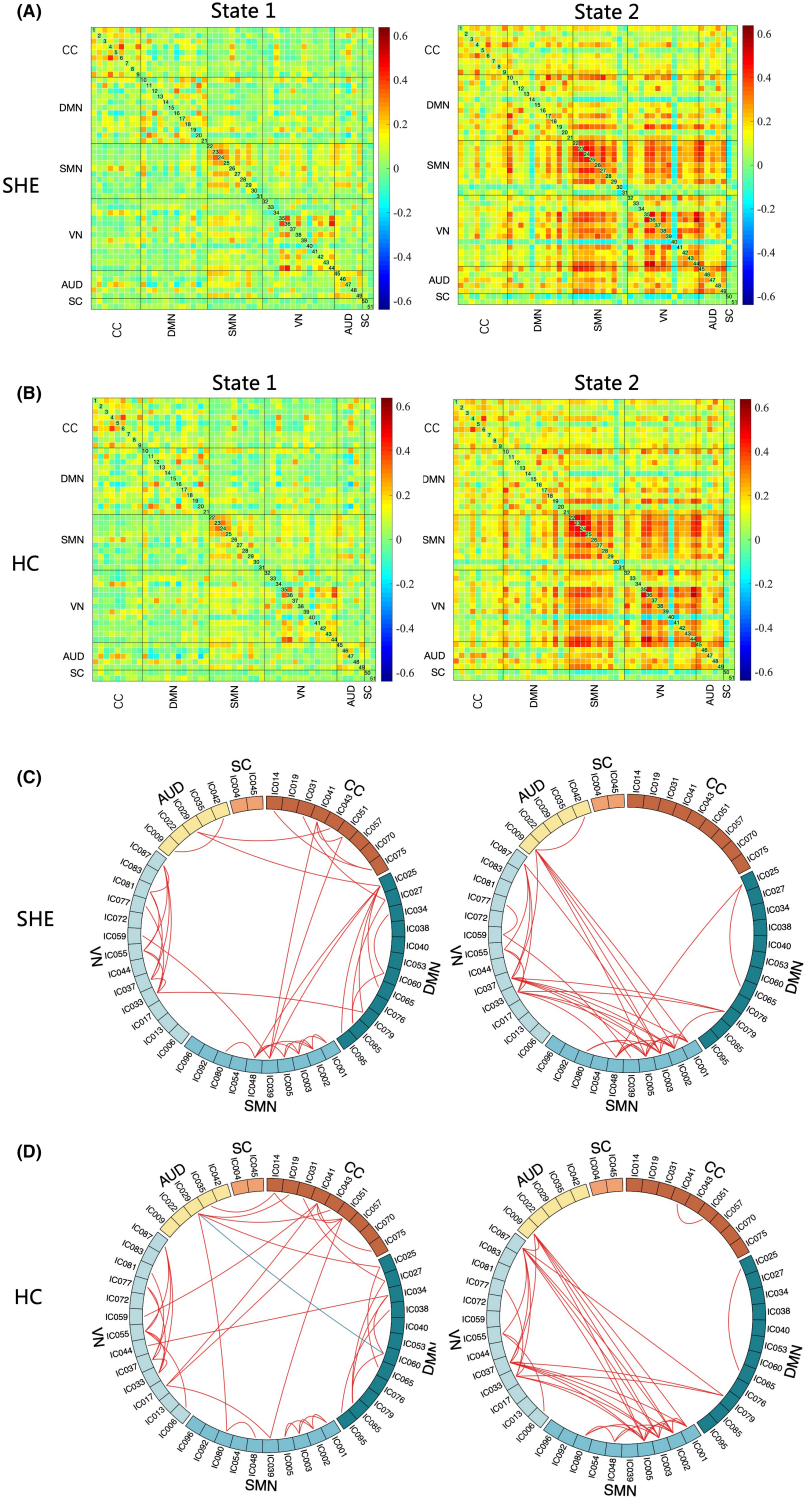
Two dynamic functional connectivity patterns of each group. *Note*: A. Cluster centroids for each state in patients with SHE. B. Cluster centroids for each state in patients with HC. C. The top 3% of the strongest FC in each state of patients with SHE. D. The top 3% of the strongest FC in each state of HC. The red lines mean positive connections, and the blue line means negative connection. AUD, auditory network; CC, cognitive control network; DMN, default mode network; SMN, sensorimotor network; SC, subcortical network; VN, visual network.

**FIGURE 5:**
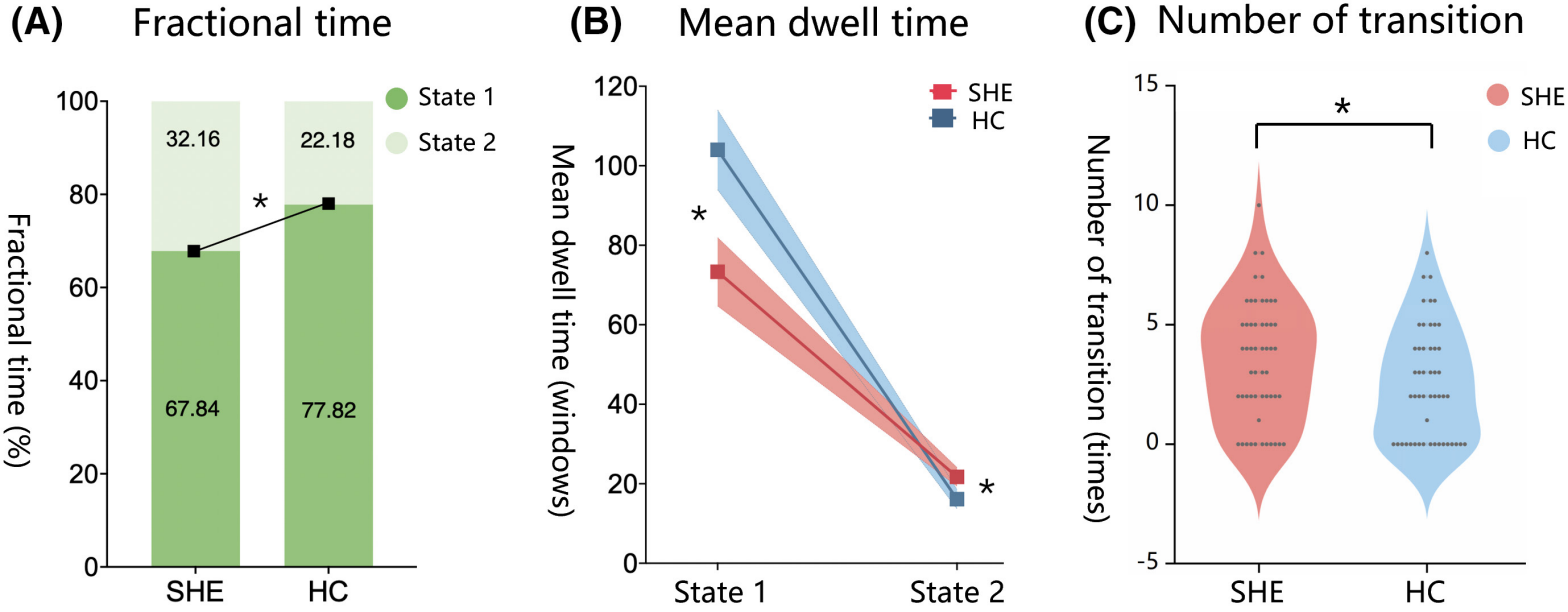
Group differences of temporal properties. *Note*: A. The difference of fractional time between patients with SHE and HC. B. The group difference of mean dwell time. (State 1: SHE = 73.32 ± 63.30%, HC = 104.00 ± 70.91%; State 2: SHE = 21.73 ± 17.40%, HC = 16.12 ± 17.29%) C. Abnormal number of transitions in patients with SHE. (SHE = 3.69 ± 2.57%, HC = 2.64 ± 2.61%) * represents significant difference (*p* < 0.05). And FDR correction was used in comparison in fractional time and mean dwell time.

After k‐means clustering, the cluster centroid of SHE and HC is shown in Figure 4. Although the dFC patterns in two states were similar, we still found the abnormal temporal properties in SHE. The incidence of SHE patients was higher in state 1 (67.84 ± 29.66%) and lower in state 2 (32.16 ± 29.66%). Moreover, the overall incidence in the HC group was 77.82 ± 25.99% in state 1 and 22.18 ± 25.99% in state 2 (Figure 4). Overall, compared with HC, patients with SHE showed greater fractional time in state 2 (*p* = 0.033, permutation test, FDR correction, Figure 5A). As for mean dwell time, both patients and HC were more likely to stay in state 1, and the mean dwell time in state 2 was relatively short. The MDT of patients and HC had statistical significance in two states (*p* = 0.018, 0.048, permutation test, FDR correction, Figure 5B). In addition, a significant difference in the number of time transitions between two groups was found (*p* = 0.020, permutation test, FDR correction, Figure 5C). The NT showed an increase in patients with SHE, which means they change from one state to another state more frequently.

When the window size was set to 30 TRs with other parameters unchanged, two dFC states were identified in the validation analysis. State 1 and state 2 under a window size of 30 TRs showed a similar dFC pattern to the ones under the window size of 22 TRs (Figure S4, Table S3). We also found consistent differences and trends in temporal properties under the two different window sizes (Table S4).

### 3.5 FC differences and Correlation analysis

**FIGURE 6:**
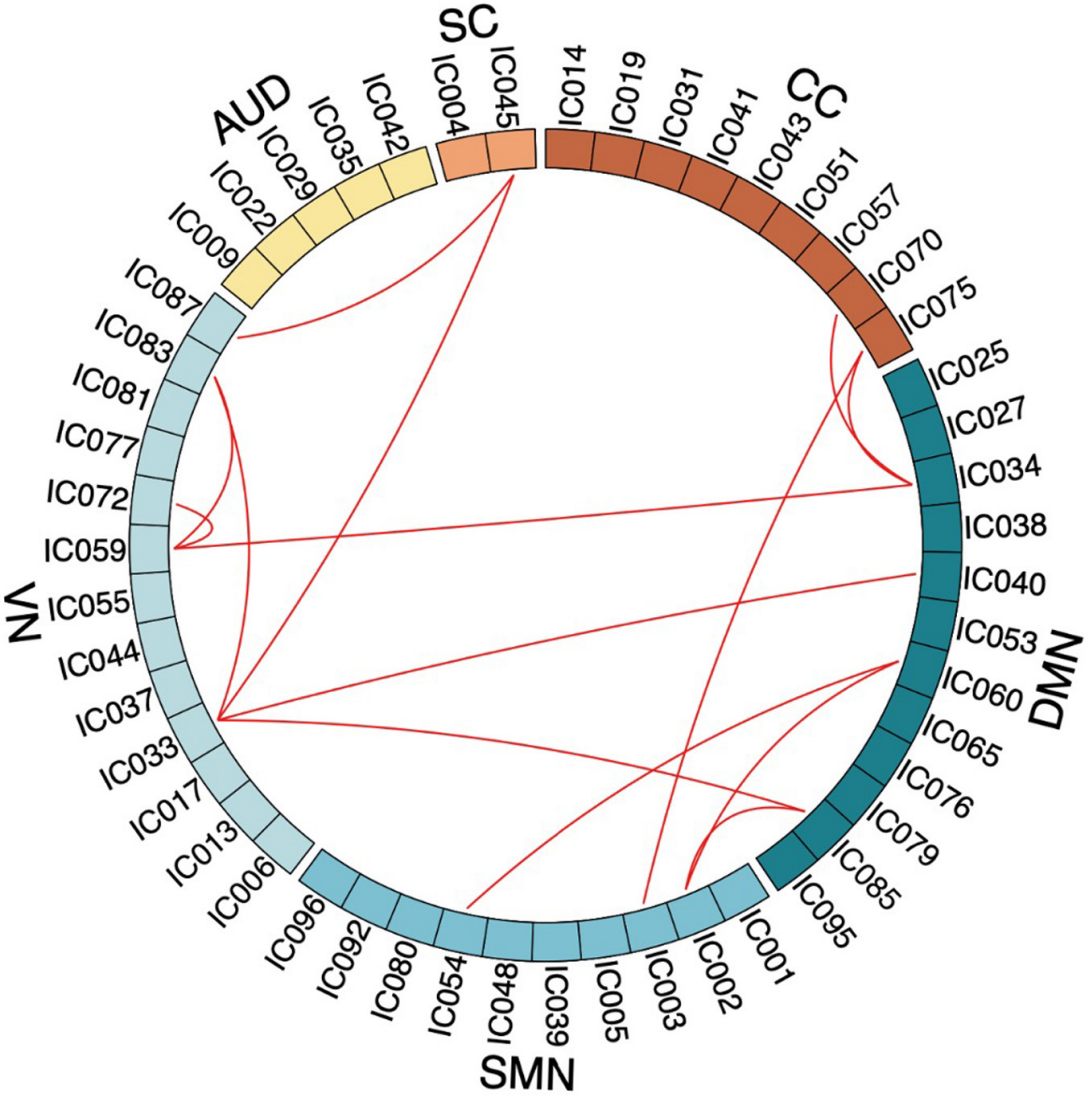
The differences of FC strength between two groups by using NBS analysis. *Note*: The red lines mean stronger connections in patients with SHE than HC in state 1.

The results of NBS found that FC group differences only occurred in state 1 (corrected *p*_component_ = 0.029, *p*_edge_ = 0.05). Patients with SHE showed stronger FC than HC in 14 pairs components (Figure 6, Table S2). They were mostly between DMN, VN, SMN, CCN and SCN, and a little within VN as well. However, we found no significant differences in state 2 between SHE and HC. We did not find any significant correlation between illness duration and temporal properties.

## 4 DISCUSSION

This study constructed the dFC matrix using GICA and sliding‐windows method and analyzed the group differences of temporal properties. Furthermore, the connectivity strength was analyzed by the NBS method. The main findings suggested as follows: there are two repetitive patterns of dFC in instinct brain. State 1 occurred frequently with weak connectivity, in which positive connection was mainly located within networks. State 2 was a less frequent and stronger connected state. The positive connections in this state were mainly between networks. The between‐group comparison of temporal properties found that patients with SHE were assigned greater windows and mean dwell time in state 2, accompanied by an increase in the number of transitions. In addition, NBS showed the connectivity strength between networks (DMN, CCN, VN, SMN, and SCN) were increased.

State 1 mainly had positive connections within CCN, DMN, VN and SMN. The decrease of fractional time in state 1 reflected the damage to the related brain regions and impairment of functional separation in patients with SHE.^28^ Among these networks, CCN and DMN were often referred to as high‐level cognitive networks. The CCN includes the fronto‐parietal regions, which is involved in attention‐related cognitive tasks, working memory and decision‐making.^33^ Its connectivity alterations may be related to the impairment of working memory in patients with SHE.^33^ As for DMN, it was activated in the resting state of brain and deactivated during executive function. Disruption of the DMN would lead to impaired consciousness and altered cognitive function in patients with epilepsy.^34^, ^35^ Several previous studies have confirmed that DMN plays an important role in epilepsy.^36^ An fMRI study on three different types of epilepsy found a general pattern of DMN impairment and subtype‐specific dysfunction.^20^ We believed that the functional impairment in DMN was mainly a manifestation of disturbed consciousness in patients with SHE.^7^, ^37^ In addition, modular analysis can measure integration within modules and separation between modules.^38^ State 1 has a higher modular pattern, where CC and DMN formed one module to be responsible for advanced cognitive functions, as well as SMN, AUD, and VN were in another module as low‐level perceptual network. This high degree of modularity means the functional separation. Decreased fractional time and mean dwell time in state 1 of patients with SHE also demonstrated reduced functional segregation of functional networks,^28^ which may facilitate the propagation of epileptic networks.

The FC characteristic of state 2 was mainly the stronger connectivity between the three networks of AUD, SMN, and VN. The abovementioned three networks participate in the process of perception and motion and have great effects on the exchange of information with the external environment.^39^ The strong connectivity between networks implies synchronicity reflected by underlying compensatory mechanisms of the instinct brain.^28^ Meanwhile, the positive connections of the perceptual network indicated an enhanced activity in communicating with external information. Furthermore, visual regions were often connected to motor areas as sensory guidance for movement, so the interaction between VN and SMN was important for motor control,^40^ which may suggest that enhanced compensatory connectivity in patients with SHE. It was widely known that hypermotor is the main symptom in SHE. Compared with HC, patients with SHE spend more time in state 2 (predominantly positive connections between networks) and less in state 1. This altered stability in network connectivity demonstrated the fragility of FC in the brain of patients with SHE.^28^, ^41^ Besides, the brain was an organ with strong adaptability. When diseases cause abnormal states in the brain, the related network would reorganize to adapt to various functional disruptions and maintain normal function.^41^ The reduction of the state occurrence means the impairments of the related network regulatory function;^18^, ^28^ thus, patients with SHE spend more time in the state dominated by positive connections between networks, which was a potential mechanism to restore the homeostasis of brain connectivity. The FC of patients with SHE in this state was unstable. Compared with HC, the number of transitions between two states was increased in SHE. The frequent transitions reflected the brain compensation to ensure functional integrity.

NBS analysis showed that patients with SHE had enhanced FC between networks in state 1 than HC, mainly between DMN and SMN, DMN and VN, and DMN and CC, suggesting that functional pattern of patients were integrated between cognitive and sensory networks. These results correspond to the FC pattern in state 2, which was another proof of temporal characteristics. As mentioned before, in the dFC state analysis, the main feature of state 1 was the positive connections within networks. The reduced incidence of patients with SHE in state 1 means that the FC within networks is damaged.^28^ At the same time, the enhanced connectivity between networks may represent a functional compensation.^18^, ^28^ It also implies an adaptation of the patients' brain to seizures, thereby stabilizing neural networks and protecting the brain from prolonged epileptic seizures.

## 5 LIMITATIONS

The main limitations of our study include two points. Most patients took antiepileptic drugs, which may have an impact on the brain function; but for ethical reasons, this confounding effect cannot be eliminated. Moreover, the sample size of our study was still small, and we look forward to collecting data in the future and using prospective studies to obtain more stable and in‐depth research evidence.^42^

## 6 CONCLUSIONS

Our study introduced information at the temporal level, enabling a more refined analysis of dynamic functional connectivity than static functional connectivity. We found that the dFC in the brain of patients with SHE tends to two different state modes, one is dominated by intra‐network connections and the other is characterized by inter‐network connections, accompanied by frequent transitions between these two states. This abnormal dFC mode reveals an adaptive mechanism in the brain after epileptic activity. The overall results will help us further understand the neural mechanism of SHE.

## AUTHOR CONTRIBUTIONS

Qiang Yue and Qiyong Gong conceived the project, Xinyue Wan and Pengfei Zhang processed the data and wrote the main manuscript. Xintong Wu mainly collected participants. Xinyue Wan, Simin Zhang, Weina Wang, Xiaorui Su, Xibiao Yang, and Qiaoyue Tan obtained the data. Shuang Li and Hanbing Shao organized the data. All authors critically reviewed the manuscript. Qiang Yue and Qiyong Gong revised the manuscript.

## CONFLICT OF INTEREST

The authors have no financial conflicts of interest to declare.

## Supporting information


Appendix S1.

## Data Availability

The data that support the findings of this study are available from the corresponding author upon reasonable request.
