# Supplementary material for: Abnormal brain functional network dynamics in sleep‐related hypermotor epilepsy
Source: CNS Neurosci Ther. 2022 Dec 12;29(2):659–68. doi: 10.1111/cns.14048 (PMC9873504; doi:10.1111/cns.14048)
Supplement: Supplementary file 1 — Appendix S1. [file CNS-29-659-s001.docx]

**Supplementary Materials For**

Abnormal brain functional network dynamics in sleep-related hypermotor epilepsy

Xinyue Wan, Ph.D., Pengfei Zhang, M.D., Weina Wang, Ph.D., Xintong Wu, Ph.D., Qiaoyue Tan, M.M., Xiaorui Su, Ph.D., Simin Zhang, Ph.D., Xibiao Yang, M.D., Shuang Li, M.D., Hanbing Shao, M.M., Qiang Yue, M.D., Ph.D., Qiyong Gong, M.D., Ph.D.


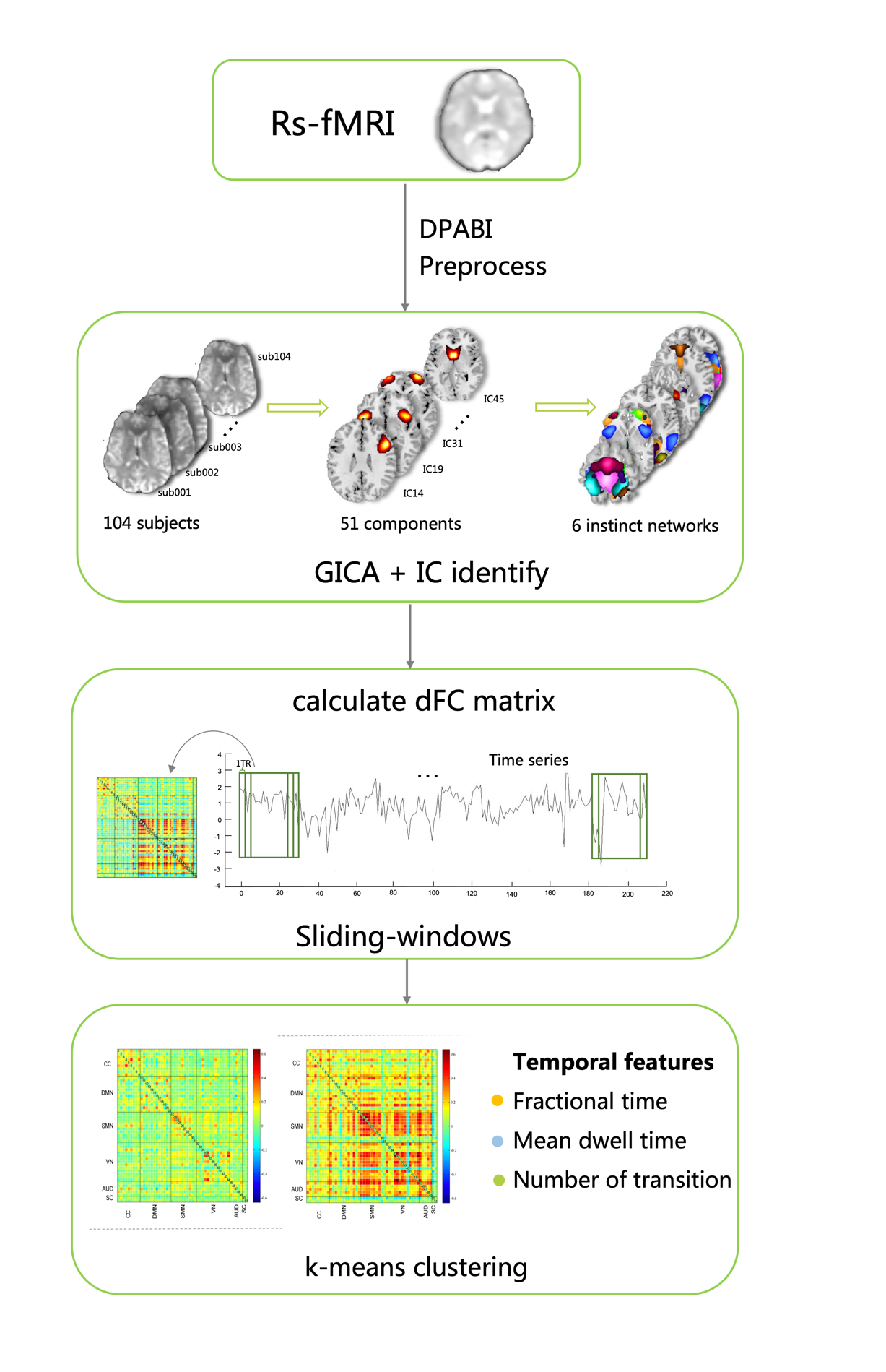


**Figure S1. The flowchart of the dFC state analysis pipeline.**


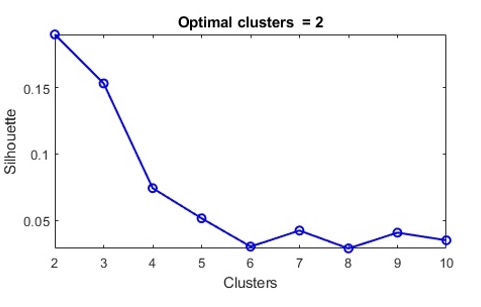


**Figure S2. The optimal K number in k-means clustering analysis.**

***Note:*** According to Siihouette statistics, it found 2 clusters in this study.

**
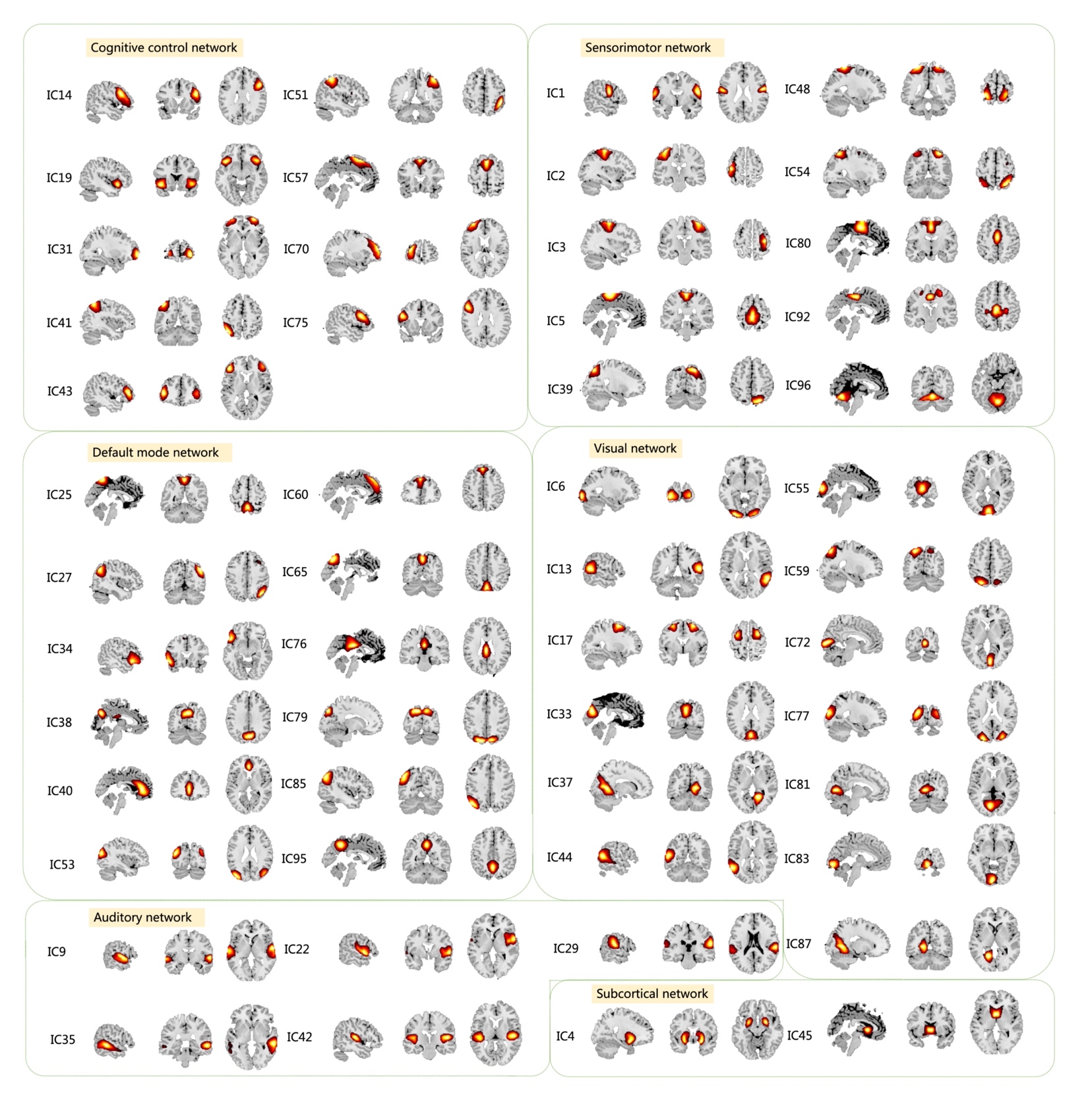
**

**Figure S3. Spatial maps for 51 independent components.**

***Abbreviations:*** IC = independent component.

**
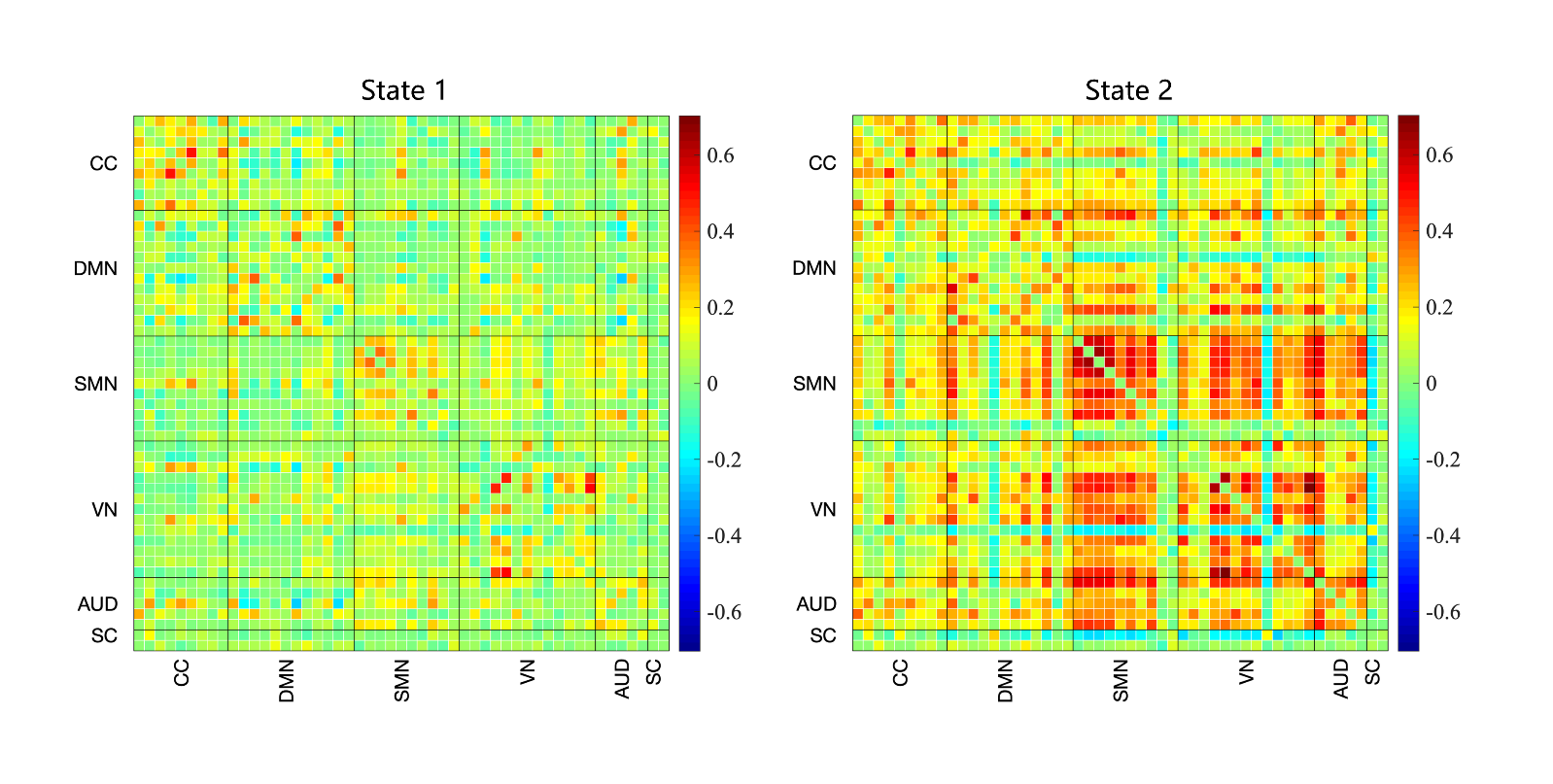
**

**Figure S4. Cluster centroids under 30 TRs windows size for each state of all participants.**

***Abbreviations:*** AUD = auditory network, SMN = sensorimotor network, SC = subcortical network, VN = visual network, CC = cognitive control network, DMN = default mode network.

**Table S1. Peak coordinates of 51 ICs.**

| **Instinct networks** | **Brain region** | **Voxels** | **T value** | **MNI coordinate** | | |
| --- | --- | --- | --- | --- | --- | --- |
|  |  |  |  | **x** | **y** | **z** |
| **Cognitive control network (CCN)** | | | | | | |
| IC014 | Precentral_R | 2459 | 25.38 | 48 | 12 | 33 |
| IC019 | Frontal_Inf_Orb_L | 1155 | 30.31 | -39 | 21 | -9 |
|  | Insula_R | 1201 | 26.69 | 42 | 18 | -12 |
| IC031 | Frontal_Sup_Orb_L | 2463 | 26.15 | 30 | 60 | -3 |
|  | Frontal_Mid_Orb_L |  |  | -36 | 57 | -3 |
| IC041 | Parietal_Inf_L | 1805 | 27.57 | -36 | -60 | 51 |
| IC043 | Frontal_Inf_Tri_L | 1494 | 26.48 | -45 | 39 | 6 |
|  | Frontal_Mid_R | 1267 | 23.78 | 48 | 45 | 3 |
| IC051 | Parietal_Inf_R | 1670 | 30.17 | 48 | -42 | 48 |
| IC057 | Supp_Motor_Area_L | 1625 | 29.71 | -3 | 18 | 57 |
| IC070 | Frontal_Mid_L | 2836 | 23.04 | -30 | 51 | 21 |
| IC075 | Frontal_Inf_Tri_L | 1786 | 25.75 | -45 | 15 | 27 |
| **Default mode network (DMN)** | | | | | | |
| IC025 | Precuneus_R | 1444 | 30.13 | 3 | -54 | 54 |
| IC027 | Angular_R | 1304 | 33.85 | 48 | -63 | 33 |
| IC034 | Temporal_Pole_Sup_L | 1459 | 25.26 | -51 | 21 | -12 |
| IC038 | Cuneus_R | 1916 | 27.59 | 12 | -69 | 30 |
| IC040 | Cingulum_Ant_L | 2341 | 28.62 | -3 | 39 | 3 |
| IC053 | Occipital_Mid_L | 1571 | 24.88 | -39 | -84 | 27 |
|  | Temporal_Mid_R | 988 | 20.76 | 51 | -69 | 21 |
| IC060 | Frontal_Sup_Medial_L | 2075 | 30.43 | 0 | 42 | 39 |
| IC065 | Precuneus_R | 1197 | 32.67 | 3 | -72 | 39 |
| IC076 | Precuneus_L | 2266 | 27.66 | 0 | -48 | 69 |
| IC079 | Occipital_Sup_L | 1542 | 23.68 | -24 | -78 | 39 |
|  | Cuneus_R |  |  | 15 | -78 | 36 |
| IC085 | Anuglar_L | 1510 | 26.54 | -42 | -66 | 33 |
| IC095 | Precuneus_L | 2055 | 32.47 | 0 | -54 | 39 |
| **Sensorimotor network (SMN)** | | | | | | |
| IC001 | Postcentral_R | 1200 | 24.57 | 48 | -12 | 33 |
|  | Postcentral_L | 1143 | 21.78 | -54 | -9 | 24 |
| IC002 | Postcentral_L | 1964 | 20.91 | -45 | -24 | 51 |
| IC003 | Postcentral_R | 1699 | 22.46 | 48 | -21 | 48 |
| IC005 | Paracentral_Lobule_R | 1682 | 27.56 | 3 | -27 | 66 |
| IC039 | Occipital_Sup_R | 2026 | 25.13 | 24 | -72 | 48 |
| IC048 | Postcentral_R | 1076 | 24.26 | 21 | -42 | 66 |
|  | Parietal_Sup_L | 1256 | 22.80 | -21 | -39 | 66 |
| IC054 | Parietal_Sup_R | 1112 | 27.14 | 30 | -60 | 57 |
|  | Parietal_Sup_L | 566 | 17.82 | -30 | -60 | 57 |
| IC080 | Cingulum_Mid_L | 2638 | 28.20 | 0 | -3 | 48 |
| IC092 | Cingulum_Mid_L | 3471 | 19.37 | -3 | -21 | 48 |
| IC096 | Vermis_6 | 2606 | 29.38 | 0 | -69 | -18 |
| **Visual network (VN)** | | | | | | |
| IC006 | Lingual_L | 991 | 23.52 | -24 | -93 | -12 |
|  | Lingual_R | 729 | 23.21 | 27 | -90 | -12 |
| IC013 | Temporal_Mid_R | 1672 | 25.06 | 57 | -54 | 12 |
| IC017 | Frontal_Sup_R | 1542 | 23.98 | 27 | 0 | 60 |
|  | Frontal_Mid_L | 1079 | 20.97 | -24 | 3 | 54 |
| IC033 | Cuneus_L | 1639 | 25.61 | 0 | -81 | 18 |
| IC037 | Lingual_R | 2445 | 23.57 | 12 | -57 | 3 |
| IC044 | Temporal_Mid_L | 2869 | 27.11 | -54 | -60 | 12 |
| IC055 | Calcarine_L | 1537 | 26.69 | 3 | -90 | -3 |
| IC059 | Parietal_Sup_L | 1287 | 29.14 | -15 | -72 | 51 |
|  | Parietal_Sup_R | 409 | 16.52 | 18 | -69 | 51 |
| IC072 | Calcarine_R | 1208 | 19.10 | 9 | -93 | 3 |
| IC077 | Occipital_Mid_R | 1256 | 24.53 | 30 | -84 | 18 |
|  | Occipital_Mid_L | 1337 | 22.22 | -24 | -84 | 18 |
| IC081 | Lingual_L | 1889 | 24.27 | -3 | -72 | 3 |
| IC083 | Calcarine_L | 1576 | 24.26 | -3 | -87 | -12 |
| IC087 | Lingual_L | 2076 | 20.26 | -12 | -60 | -3 |
| **Auditory network (AUD)** | | | | | | |
| IC009 | Temporal_Sup_R | 1387 | 21.63 | 63 | -21 | 9 |
|  | Temporal_Sup_L | 1381 | 21.47 | -60 | -24 | 6 |
| IC022 | Temporal_Sup_R | 2385 | 21.45 | 57 | -3 | 3 |
| IC029 | Temporal_Sup_R | 1152 | 26.70 | 60 | -27 | 15 |
|  | Temporal_Sup_L | 725 | 18.45 | -60 | -33 | 18 |
| IC035 | Temporal_Mid_L | 1999 | 28.72 | 60 | -33 | -6 |
| IC042 | Temporal_Sup_R | 1409 | 23.84 | 51 | -21 | 9 |
| **Subcortical network (SCN)** | | | | | | |
| IC004 | Amygdala_R | 1244 | 28.47 | 27 | 3 | -12 |
|  | Putamen_L | 1268 | 26.26 | -27 | 3 | 0 |
| IC045 | Caudate_L | 1580 | 29.38 | -9 | 15 | 0 |

***Abbreviations:*** IC = independent component, L = left, R = right, Inf = inferior, Orb = orbital, Sup = superior, Mid = middle, Tri = triangularis.

**Table S2. The information about stronger FC from NBS analysis.**

| **Functional connectivity** | ***p* value** |
| --- | --- |
| **Inter-network** |  |
| IC34-IC70 | 0.005 |
| IC34-IC75 | 0.006 |
| IC2-IC60 | 0.008 |
| IC2-IC85 | 0.004 |
| IC3-IC75 | 0.001 |
| IC54-IC60 | 0.004 |
| IC33-IC40 | 0.005 |
| IC33-IC85 | 0.009 |
| IC34-IC59 | 0.006 |
| IC33-IC45 | <0.001 |
| IC45-IC87 | 0.009 |
| **Intra-network** |  |
| IC59-IC72 | <0.001 |
| IC33-IC83 | 0.009 |
| IC59-IC83 | 0.001 |

***Note****:* It showed the stronger FC in patients with SHE than HC in state 1.

***Abbreviations:*** IC = independent component.

**Table S3. The correspondence between pairs of centroids in dynamic functional connectivity states under the two different window sizes (22 TRs and 30 TRs).**

| **Window size**  **= 30TRs** | **Window size = 22TRs** | |
| --- | --- | --- |
|  | State1 | State2 |
| State1 | *r* = 0.999 (*P* < 0.001)* | *r* = 0.691 (*P* < 0.001) |
| State2 | *r* = 0.715 (*P* < 0.001) | *r* = 0.998 (*P* < 0.001)* |

***Note****:* * represents the high similarity of state centroids under the two different window sizes.

**Table S4. Temporal properties of dynamic functional connectivity states with 30 TRs window size in patients with SHE and HC.**

| **Temporal properties** | **States** | | **SHE** | | **HC** | | ***p* value** |
| --- | --- | --- | --- | --- | --- | --- | --- |
| Fractional windows | State 1 | 64.69±32.78% | | 76.60±28.95% | | 0.023 (FDR) | |
|  | State 2 | 35.31±32.78% | | 23.40±28.95% | | 0.023 (FDR) | |
| Mean dwell time | State 1 | 84.29±66.57 | | 108.48±65.76 | | 0.045 (FDR) | |
|  | State 2 | 37.48±45.02 | | 20.86±23.91 | | 0.016 (FDR) | |
| Number of transitions | - | 2.26±2.01 | | 1.78±1.79 | | 0.178 | |

***Note****:* Two dynamic functional connectivity (dFC) states were identified in validation analysis with a window size of 30 TRs. State 1 was characterized by widely sparse weak connectivity and state 2 showed the relatively stronger connectivity. Values were shown as mean ± SD. Nonparametric permutation tests (10000 iterations) were applied (p < 0.05, using a false discovery rate (FDR) correction) to calculate differences in temporal properties between two groups.

***Abbreviations:*** SHE = sleep-related hypermotor epilepsy; HC = healthy control.
